# Surviving a classic heat stroke/hyperthermia > 42 °C – a case report

**DOI:** 10.1186/s12245-024-00705-2

**Published:** 2024-09-10

**Authors:** Sonja Verena Schmidt, Jannik Hinzmann, Anna Stammler, Paula Wilhelms zu Bickern, Elisabete Macedo Santos, Marcus Lehnhardt, Christoph Wallner

**Affiliations:** 1 Department of Plastic Surgery, BG University Hospital Bergmannsheil Bochum, Ruhr-University Bochum, Bürkle de la Camp-Platz 1, 44789 Bochum, Germany; 2 Department of Anesthesiology, BG University Hospital Bergmannsheil Bochum, Ruhr-University Bochum, Bochum, Germany

**Keywords:** Hyperthermia, Heat stroke, Burns, Facial reconstruction, Case report

## Abstract

**Introduction:**

Classic heat stroke is a severe trauma which can lead to multi-organ dysfunctions and is associated with a high mortality.

**Case presentation:**

In this case report we present a 73-year-old patient with a classic heat stroke. His initial core body temperature was over 42 °C and he had a GCS of 3. Due to severe burn injuries the patient was transferred to a specialized burn center. The patient developed different organ failures and showed a prolonged course on the intensive care unit. Although the patient demonstrated different impaired organ systems, he recovered completely after receiving painstaking supportive therapy.

**Conclusions:**

This is a rare case of a patient who fully recovered after a heat stroke with a temperature over 42 °C and severe sequelae.

## Introduction

Heat stroke (HS) is the most severe form of heat-related illnesses and defined by a core body temperature > 40 °C / 104 °F and an impaired central nervous system, respectively consciousness [1]. The condition can be classified as classic heat stroke (CHS) induced by passive heat exposure or as exertional heat stroke (EHS) which occurs during physical exercise [2]. HS is a serious occurrence that can result in various complications concerning multiple organ systems and ultimately pose a threat to life. Under intensive care, classic heat strokes reach mortalities of up to 63.2% [3] du to multi-organ dysfunctions such as rhabdomyolysis, acute renal and hepatic failure, coagulopathy, myocardial injury, or acute respiratory distress syndrome [4]. The mechanism of heat strokes’ sequelae is based on the cytotoxic effects of an elevated core body temperature and a consecutive inflammatory response [5].

Different laboratory biomarkers have been identified as significant indicators for mortality of exertional as well as classic heat strokes. They are indicative for different organ failures, for example albumin, alanine and aspartate aminotransferase, lactate and prothrombin time for liver failure or creatinine and blood urea nitrogen for renal impairment. Other prognostic relevant values are platelet count, neutrophils, and lymphocytes [6].

In the present case, we introduce a patient with a core body temperature exceeding 42 °C who suffered from multiple organ disorders. However, he ultimately survived with only minimal residual impairments after several weeks of intensive care treatment.

## Case presentation

Given the intricate nature of the case, the presentation is divided into two distinct paragraphs: one addressing the intensive care treatment and another focusing on the plastic surgical management of the burn wounds.

A 73-year-old male patient was found unconscious and unresponsive in a sauna with a room-temperature of 80 °C. When paramedics arrived, the patient was found to be put into stable side position and presented a GCS Score of 3. Initially, paramedics found a centralized patient with a not measurable core body temperature > 42 °C (thermometer was limited to measure only under 42 °C / 107,6 °F). After intubation and administration of crystalloid volume for cooling purpose, the patient was administered to the emergency department for further evaluation. Cooling in the form of immersion in cold water or any other cooling method has not been carried out. The patient had a medical history of 1-vessel CAD with 50% medial RCA stenosis, gastric carcinoma, arterial hypertension, lumbar disc herniation, depression, benign prostatic hyperplasia and nitro intolerance. Medications included ASS, Trevelor, Durogesic, metoclopramide and transepidermal fentanyl.

An evaluation in the emergency room revealed an intubated patient, with an erythroderma over the whole body, a blood pressure of 136/74 mmHg without administration of catecholamines and a heart rate of 139 beats per minute. The temperature was 39.8 °C after arriving at the hospital and the administered volume until this time point was 1000 ml of crystalloid fluid.

Because of the unconsciousness, a CT head scan was performed instantaneously which did not show any signs of herniation but an edema of the parietal cortex which presented due to narrow external liquor spaces. Thus, 8 mg of dexamethasone were administered. Subsequently, the patient was administered to the intensive care unit and received basic treatment including fluid resuscitation for further cooling purpose.

The next day, due to demarcation of severely burned areas, the patient was transferred to a specialized burn center for further therapy. At the time of admission the patient was still intubated, sedated and hemodynamically stable without administration of catecholamines. In a neurologic examination, no hints of focal neurologic deficits could be detected. Pupils were narrow under therapy of opioids but reacted promptly in direct and indirect testing. For the burns, the Parkland-Baxter formula was used and a volume requirement of 2 L was calculated. During the first day in the burn unit, the patient received a total of 4 L of crystalloid due to volume depletion and the need for higher volume resuscitation due to extracellular shift and capillary leak. Therefore, a total fluid resuscitation of 9–10 L within the first 48 h of trauma was carried out.

In the laboratory, the patient showed signs of beginning liver failure, coagulopathy and DIC already on day 1 after trauma when arriving at the burn center (Table 1). He had mild bleeding over the gastric tube and minimal petechial signs at the lower extremity. The home medication with ASS was paused because of thrombocytopenia and vitamin K supplementation was initiated due to impaired coagulation (Quick/ INR value). Additionally, fibrinogen as well as albumin were supplemented due to extremely low laboratory values. Contemporaneously, a massive hypophosphatemia with a lowest value 4 days after trauma, was treated for several days. Furthermore, we observed an initial increase of creatine kinase to 3022 U/L on the first day which decreased consciously in the following days and was interpreted as indicator for muscle death due to extreme heat exposure.

**Table 1:** Laboratory values

Laboratory values	Day 1 12 p.m.	Day 1 8 p.m.	Day 2 4 a.m.	Day 2 1 p.m	Day 2 8 p.m.	Day 3 4 a.m.	Day 4 4 a.m.	Day 5 4 a.m.
Cr mg/dl	1.1	1.0	1.0	1.0	0.9	0.9	0.8	0.6
BUN mg/dl	54.8	54.8	58.2	56.9	60.8	63.1	55.0	58.8
Pro g/dl	4.1	3.6	3.5	3.5	3.1	3.1	3.4	3.7
Alb g/dl	2.4	2.0	2.2	2.4	2.2	2.4	2.6	2.6
GOT U/l	261	383	1222	1877	1985	1695	882	450
GPT U/l	120	248	909	1443	1678	1599	857	1184
y-GT U/l	29	28	28	25	25	25	29	141
LDH U/l	769	750	1351	1638	1484	1003	367	292
Bili mg/dl	0.5	1.1	1.8	2.0	2.1	2.4	5.2	6.0
CK U/l	3022	2323	1592	1352	1264	1018	503	415
CK-MB U/l	n.m.	71	n.m.	n.m.	n.m.	n.m.	n.m.	n.m.
Trop pg/ml	513.6	334.1	n.m.	n.m.	n.m.	n.m.	n.m.	n.m.
Hb g/dl	12.7	10.9	10.4	9.2	9.5	9.2	8.9	9.7
Hkt %	36.9	32.0	31.8	28.2	29.2	28.0	25.6	29.2
WBC /nl	8.6	8.9	9.2	6.9	7.0	7.1	5.9	9.1
PLT /nl	96	62	65	47	46	45	48	77
Quick %	30	15	15	15	15	16	41	64
INR	2.4	4.6	4.5	4.6	4.6	4.2	1.9	1.3
Fib mg/dl	36	105	92	245	208	186	223	327
ATIII %	133	38	32	24	20	16	18	27
D-Dim ug/ml	n.m.	> 4	n.m.	> 4	> 4	> 4	n.m.	n.m.
Lac mmol/l	3.9	1.9	1.8	1.7	1.1	0.9	1.2	0.7

Overview over the most relevant laboratory values within the first days after trauma.

On the third day at the burn unit, the patient showed an unspecific awakening response after reducing the sedation, he opened his eyes spontaneously and bit on the tubus but the reactions were not adequate and after an incidence of vomiting the sedation needed to be deepened again.

During the following days, further weaning trials were attempted: the patient presented cough and swallow reflexes but was extremely agitated with elevated blood pressure and no adequate or orientated reaction.

**Fig. 1:**
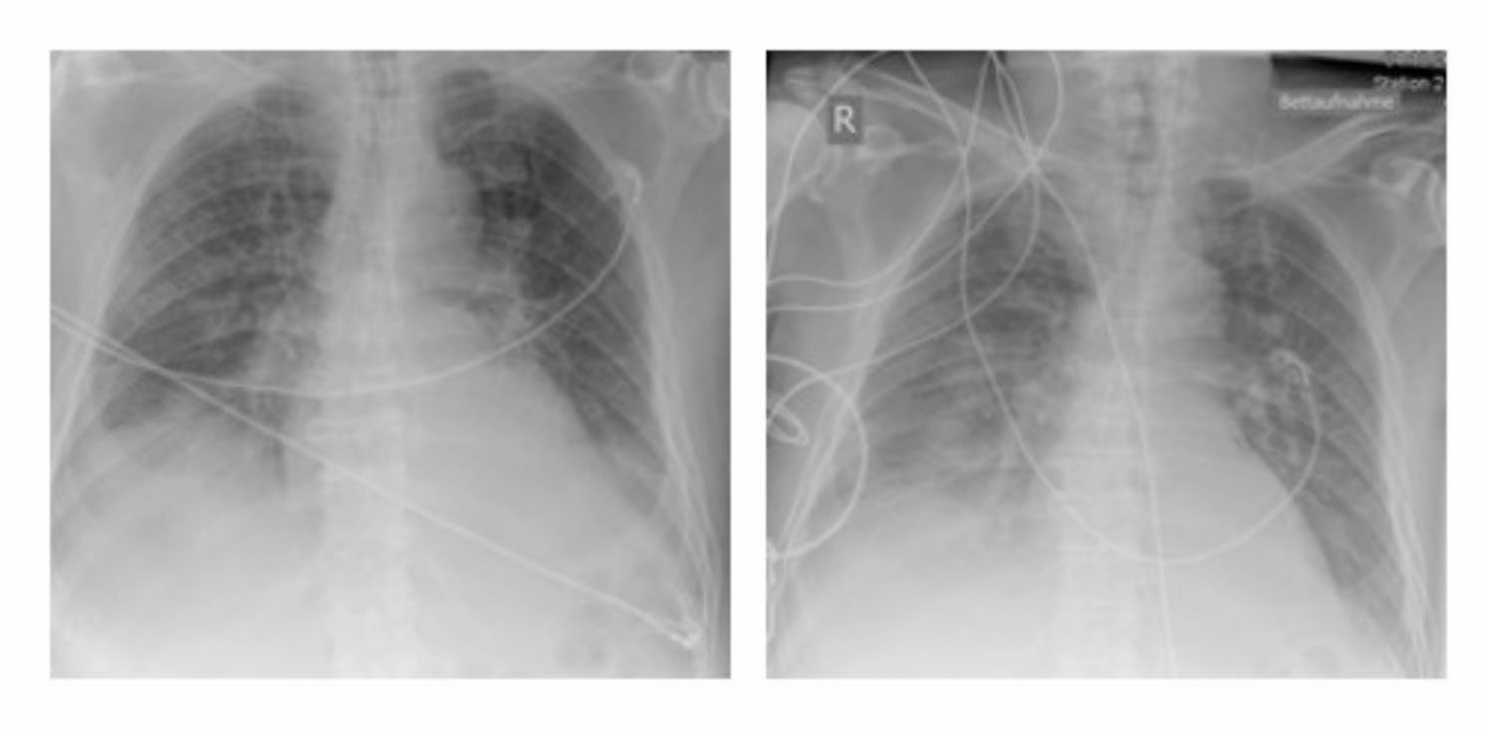
Chest x-rays during ICU stay Left: day of admission to burn unit (1 day after trauma), small dorso-basal effusions on both sides. Patchy increase in markings in the right upper field. Right: day 10 after trauma, clinically deteriorating pneumonia, Small dorsal pleural effusion on the right, with suspected infiltrates on the right basal side.

Furthermore, the patient developed a fever with temperatures > 40 °C/ 104 °F as sequela of a septic/ infectious event. With the help of physical cooling in form of cool packs and volume substitution the temperature decreased properly. Subsequently, staphylococcus aureus was detected in tracheal secretion, and an antibiotic therapy with flucloxacillin according to sensitivity testing was established. Under antibiotic therapy, the patient still presented episodes of fluctuating fever during the following days and a slight increase in CRP (11,3 mg/dl) and WBC (12,1/nl). After a few days, Klebsiella oxytoca was also proven in the tracheal secretion and because of still extensive putride secretion, antibiotic therapy was supplemented with meropenem. (Fig. 1)

Concerning the mental status, the patient did not show adequate reactions within several days, therefore a physostigmine trial was performed to reverse possibly remaining anesthesia effects [7]. After applying physostigmine over around 8 h, the patient developed more adequate and determined reactions and followed the corresponding prompts. It was administered for a total of 24 h and afterwards an administration of amantadine was performed for 3 days as amantadine is known to enhance arousal due to dopamine effects [8]. During that time, consciousness was fluctuating but finally, the patient was fully orientated and could answer “yes/no” questions properly. Also, there was a constant improvement regarding ventilation pressure and tracheal secretion, so that the patient could be weaned and extubated on the 8th day after the trauma.

Within the next days, the patient presented a right sided hemiparesis and hemihypesthesia, so that the differential diagnosis of thermal cortical degeneration of the parietal cortex was made by neurologists. Furthermore, he also presented signs of dysphagia. Under extensive ergo- and physiotherapy, the neurologic impairments ameliorated continuously over several days.

Eventually, the patient could be transferred to the general ward on the 16th day after the initial trauma. The patient was hemodynamical and respiratory stable but still needed physiotherapy due to muscle weakness. There were no remaining neurological issues.

**Fig. 2:**
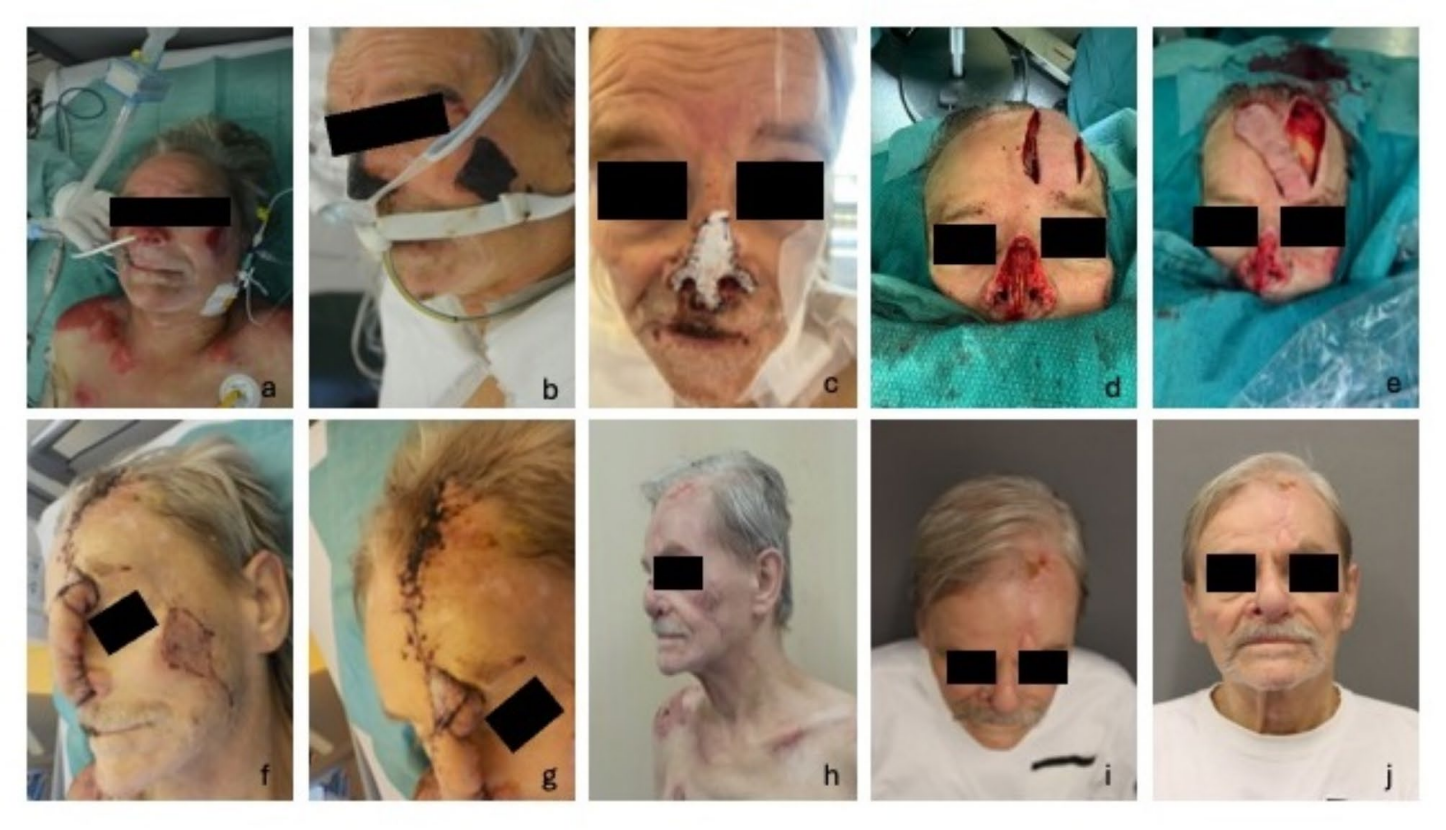
Overview of the reconstructive course of the nasal full-thickness burn (**a**) first day after trauma, deep red burn, no adequate recapillary time. (**b**) 14 days after trauma, burns at nose and cheek demarcated as dry necrotic tissue. (**c**) after first surgery and coverage of debrided nose with epigard. **d** and **e**) intraoperative images during reconstruction of nose with paramedian forehead flap. **f** and **g**) images of paramedian forehead flap before division of the pedicle. **h**) image 2 weeks after pedicle division. **i** and **j**) final result two weeks after last surgery.

Concerning the burn trauma an orientation examination was performed after admission to the burn unit. A total burned body surface area of approximately 5% was evaluated, located at the neck, thorax, both shoulders and the nose. After careful mechanical debridement of the wounds, they were classified as deep dermal/ full-thickness burns. Considering the heat stroke, elevated hepatic parameters, and the patient’s compromised general condition, we opted not to proceed with enzymatic debridement using Nexobrid^®^. This decision was made to reduce the potential risk of exacerbating the inflammatory response, respectively inducing additional metabolic stress. Wounds were reevaluated every two days and treated with aseptic fat gaze dressings. During the following days, the wounds demarcated as full-thickness burns and appeared dry necrotic. Therefore, after stabilization of the patient, first surgery was performed 14 days after the initial trauma. An epifascial necrosectomy of all wounds was performed. At the left shoulder, an advancement and primary closure was carried out, at the thorax and right shoulder a biodegradable synthetic dermal substitute, NovoSorb^®^ BTM, was applied after necrosectomy. At the face, Epigard^®^ was used as temporal coverage for the cheek and the nose. After another 10 days the second surgery was performed. A skin transplantation was conducted for the burns on the shoulder and cheek, a split-thickness skin graft for the shoulder and a full-thickness skin graft for the cheek. Advanced reconstruction of the nose was necessary due to the required removal of not only skin and soft tissue but also cartilage during the initial surgery. Therefore, a cartilaginous rib graft was harvested and shaped according to a previously created template. Subsequently, a paramedian forehead flap was performed for reconstruction of skin and soft tissue. Therefore, the pedicle with the supratrochlear artery was identified using a color doppler and marked as far as possible. 3 weeks after performing the paramedian forehead flap, resection of the pedicle was executed. Additionally, the patient presented a wound healing disorder at the forehead, where the flap lifting was performed. During surgery, only debridement with a sharp curette was executed as the wound ground presented well-perfused and therefore, conservative therapy was aimed for. The wound at the forehead healed adequately during a time of several weeks. After 3 months, the patient complained of insufficient soft tissue at the right nostril and therefore, because of the exposure of the mucous membrane to air, he experienced irritation and increased secretion. Thus, another surgery was performed in which the right nostril was reconstructed via a local rotation flap. After surgery the symptoms alleviated satisfactory, but the patient established a habitual “raising of the nose” which still bothers him. (Fig. 2)

In conclusion, the wounds healed adequately, remaining scars still get treated with regular scar massage and creaming with fatty externa. The patient is progressively content with the appearance of his face and especially nose but still faces some insecurities and psychological obstacles after the trauma and the prolonged healing process.

## Discussion

Heat stroke, in particular classical heat stroke, is an extremely dangerous condition with a relatively high mortality of over 60%. Cases of patients who survived a hyperthermia over 42 °C are extremely rare [9]. Our patient was found in the sauna, therefore presented with a classical heat stroke, and showed an initial core body temperature over 42 °C.

Heat strokes with such high core body temperatures are mostly followed by failure of multiple organs. Liver failure, AKI and shock are considered the most common consequences which lead to the high mortality rates of heat strokes [10].

**Fig. 3:**
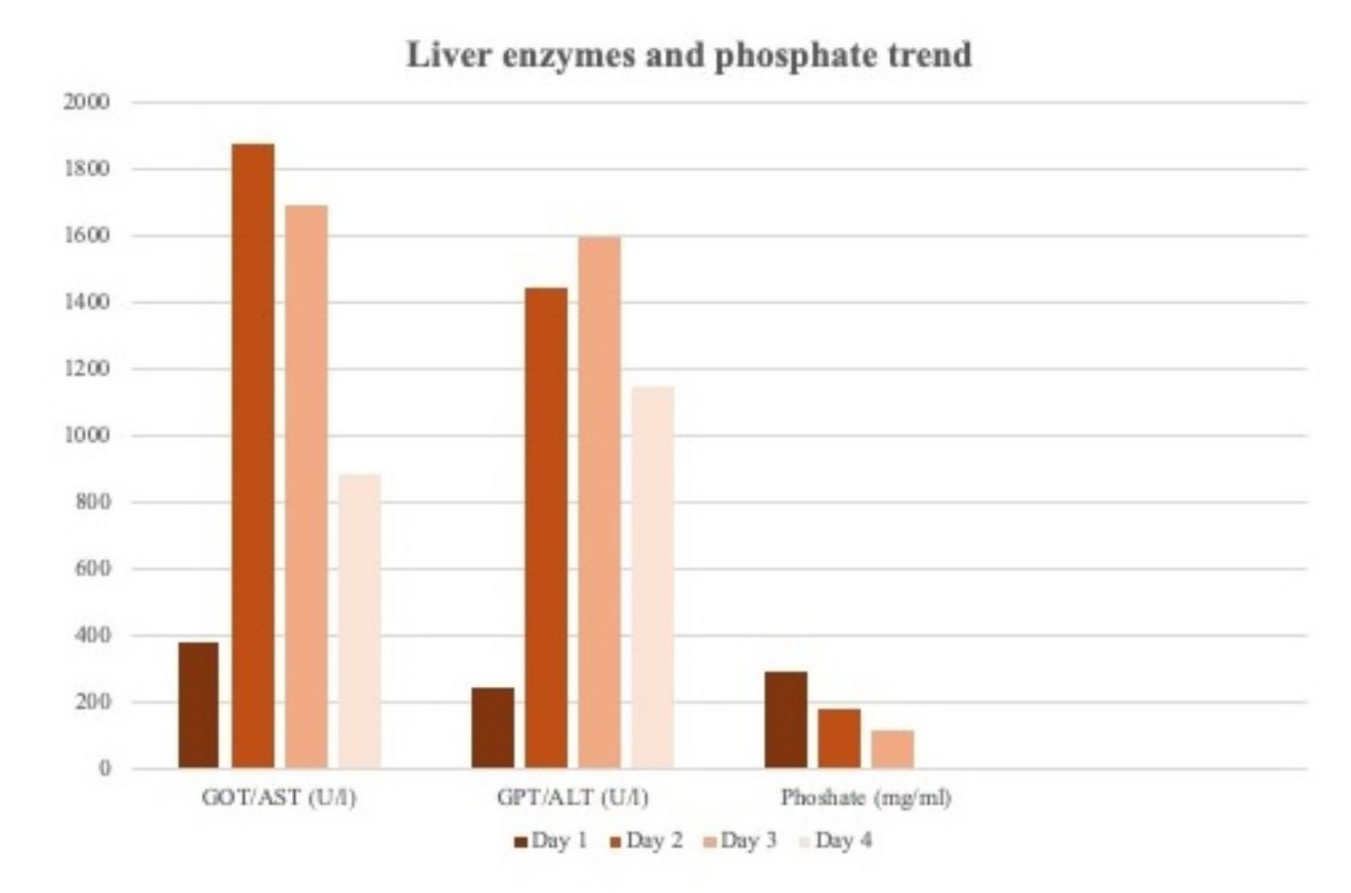
Overview of liver enzymes and phosphate levels within the first 4 days after trauma Liver enzymes have their peak on second days whereas the phosphate level presented its lowest on the fourth day after the heat stroke

In the present case, the patient presented signs of acute liver failure already the first day after the trauma. Liver enzymes were increasing progressively over the first three days and further indicators such as impaired coagulation were registered. In our case, symptomatic therapeutic measures in form of substitution of coagulation factors and amino acids were performed. Liver failure is known to be one of the most frequent incidences of organ failure after heat stroke [11]. Cases of classic and exertional heat stroke with acute liver failure as a leading symptom have been described [12]. Serum alanine aminotransferase is reported to rise within 30 min after the event of the heat stroke and to reach its peak within the first 3–4 days. These observations match the findings in our case as we detected elevated liver enzymes already in the first laboratory measurements which reached a peak on the third day after the trauma. Liver failure is a common organ dysfunction in heat strokes and elevated liver enzymes count as an important predictive value [13]. Furthermore, hypophosphatemia on admission is described to predict the occurrence of liver failure and should be monitored for three days [11]. Hypophosphatemia is also a valid prognostic factor for outcomes of patients with liver failure after partial liver resection [14]. Prolonged and severe hypophosphatemia can lead to brain and myocardial dysfunction, defects of erythrocyte and platelet structure and renal tubular defects [15]. Also, in this case we could observe severe hypophosphatemia although we discovered the lowest peak to develop with delay in comparison to the peak of the liver enzymes. Therefore, phosphate in patients suffering from heat stroke should be monitored regularly and a substitution of phosphate should be considered and reevaluated daily [16]. (Fig. 3)

Further highly relevant and severe consequences implicate impairment of the central nervous system. Especially in cases with a temperature over 41 °C, neurologic damage in form of coma or seizures marks a serious condition [17]. The most common neurologic long-term sequelae are cerebellar ataxia, dysarthria, cognitive disorders, and anterograde amnesia, although studies mostly focus on exertional heat strokes [18]. In our case the patient presented signs of hemiparesis and -hypesthesia of the right upper limb. The differential diagnosis of a thermal cortical degeneration of the left parietal cortex as a consequence of the classic heat stroke versus a middle cerebral artery infarct was discussed. Hyperthermia can lead to disruption of the blood-brain barrier which can result in cerebral edema and neuronal damage. In autopsies of patients who have died from heat stroke, cerebral edema was a common finding [19]. Cases of patients who presented findings of cerebral edema in initial radiographic imaging are rare and there are almost no cases in which patients survived this condition or recovered without residues [20]. Our patient initially presented cerebral edema with parietal emphasis and therefore received 8 mg of dexamethasone as well as continued forced cooling. The remaining sequela in form of hemiparesis and -hypesthesia improved entirely within a time period of several weeks.

Another important aspect to mention in this case is the management of cooling. The evidence for different cooling methods is still insufficient. For exertional heat stroke, ice-water immersion seems to be very effective. For non-exertional heat stroke, such as classic heat stroke, evaporative plus convective cooling is recommended by several studies [21]. The literature also mentions cooled intravenous fluids as effective in heat stroke. In this case, the patient had a classic heat stroke and was cooled with intravenous fluids. Unfortunately, we have no records of other cooling techniques, but in this case cooling with strategically applied ice packs (e.g. neck, axilla, groin) could have been discussed. However, as immersion is more commonly recommended for exertional heat stroke and cooling devices are not recommended as primary treatment, the treatment of this patient can be judged as sufficient, as cooling with intravenous fluids worked well and quickly. Nevertheless, it is very important to be aware of different cooling techniques and to carefully reassess the cooling process.

## Conclusion

In conclusion, this case demonstrates different complications following a classic heat stroke. The patient initially presented a core body temperature over 42 °C and received intense cooling measures. Although he suffered from acute liver failure, severe hypophosphatemia, neurologic impairment, and severe burns as well as consecutive septic events, the patient recovered fully due to meticulous intensive care treatment. This case emphasizes the importance of rapid cooling after heat strokes and a complex and advanced intensive care treatment for these critically ill patients.

## Data Availability

No datasets were generated or analysed during the current study.
